# Bilateral posterior hip dislocation associated with right Pipkin II fracture: A case report

**DOI:** 10.1016/j.ijscr.2019.07.017

**Published:** 2019-07-19

**Authors:** Abderrahim Zaizi, Hicham A. Benomar, Mohammed Reda Fekhaoui, Talal Grimi, Monsef Boufettal, Mohamed S. Berrada

**Affiliations:** a Department of Orthopaedic Surgery & Traumatology II, Mohamed V Military Hospital, Faculty of Medicine and Pharmacy, Mohamed V University, Rabat 10100, Morocco; b Department of Orthopaedic Surgery & Traumatology, Ibn Sina Hospital, University Mohamed V, Rabat, Morocco; c Department of Anatomy, Faculty of Medicine and Pharmacy, University Mohamed V, Rabat, Morocco; d Head of Orthopaedic Surgery & Traumatology, Ibn Sina Hospital, University Mohamed V, Rabat, Morocco

**Keywords:** Hip dislocation, Bilateral, Femoral head, Fracture, Pipkin

## Abstract

**Background:**

Bilateral posterior hip dislocations are very rare injury, requiring a very hight trauma energy.

**Presentation of the case:**

We present a case of 40-year-old male who sustained bilateral posterior hip dislocation with associated right femoral head fracture Pipkin type II following a hight energy trauma without neurovascular deficit. A prompt closed reduction was made, it was successful in lift hip but incomplete in right one, therefore, an open reduction was indicated, performed through a modified Hardinge approach permitting internal osteosynthesis with two Herbert screws.

**Discussion:**

Posterior hip dislocations are an orthopaedic emergency that must be reduced within 6 h to avoid sciatic nerve compression and avascular necrosis (AVN). They are most often associated with femoral head fractures commonly known as Pipkin’s fractures, that need anatomic reduction and osteosynthesis through posterior approaches, rarely external or anterior approaches.

**Results:**

Anatomic and functional results were good at two years follow up excepting a right hip non-bridging heterotopic ossification; the patient returned to his work normally without any functional sequelae.

## Highlights

• Bilateral posterior hip dislocations are very rare injury, resulting from high energy trauma.
• After elimination of vital emergencies and radiological assessment, reduction must be rapid within 6 h.
• Post reduction CT-scan is essential to analyze the femoral head and the hip joint congruity.
• In case of irreducibility or associated Pipkin’s fracture, an open approach is indicated.
• The main complications are avascular necrosis, heterotopic ossification, and posttraumatic osteoarthritis.

## 1 Introduction

Bilateral posterior hip dislocations are very rare injury, requiring a hight-energy accident during a dashboard mechanism. Associated femoral head fracture are frequent, they worsen the prognosis because of increased risk of osteoarthritis and avascular necrosis. Below we describe a case of young adult who underwent bilateral hip dislocation associated with Pipkin II femoral head fracture, we detailed the treatment protocols, short and long-term follow-up with literature review.

This work was reported in line with the SCARE criteria [1].

## 2 Case report

**Fig. 1:**
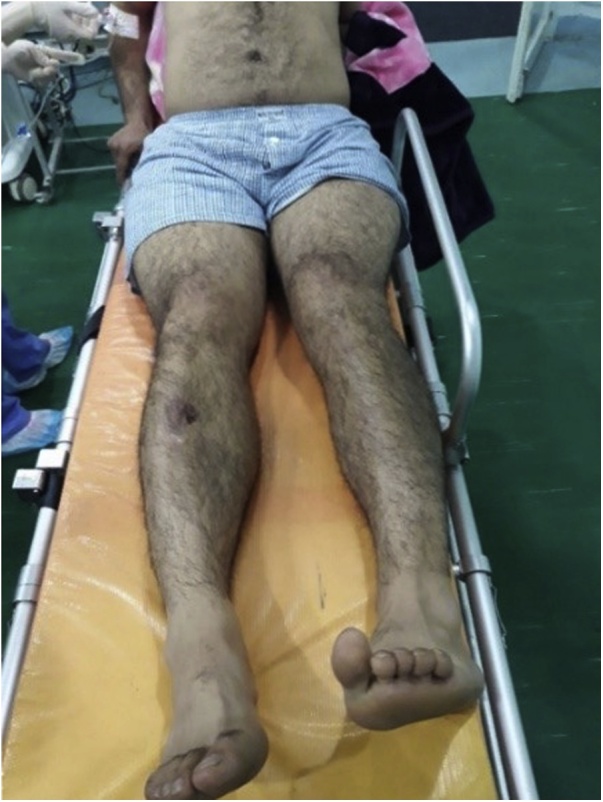
Clinical photograph of the patient, with hip flexion and internal rotation.

**Fig. 2:**
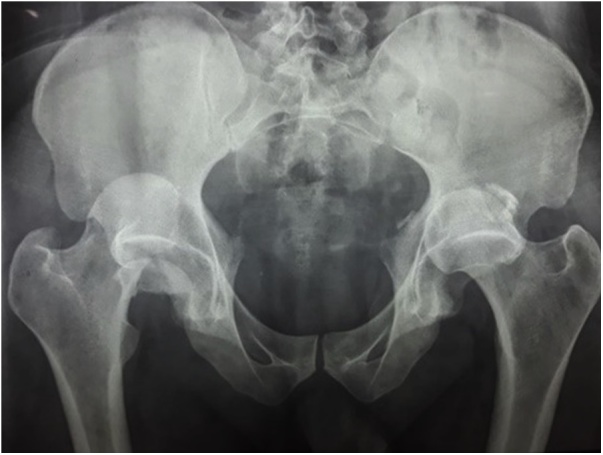
Anteroposterior radiograph of the pelvis showing bilateral posterior hip dislocation with right Pipkin type II fracture.

**Fig. 3:**
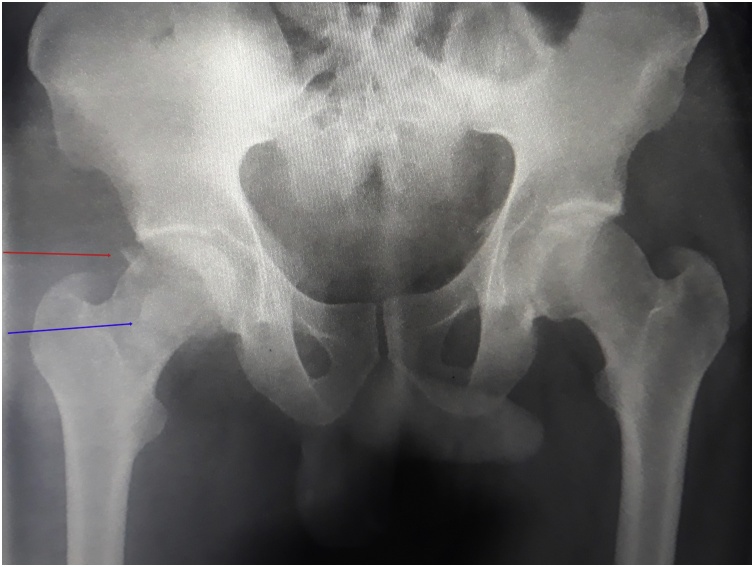
Immediate pelvic X-ray showing a non-reduction of right hip because of a non-reduced Pipkin II fragment (blue arrow) with a small superior fragment (red arrow).

**Fig. 4:**
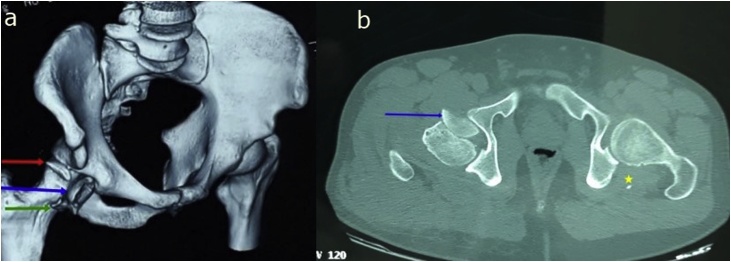
3D CT-scan (a) with transversal view (b) showing a non-anatomic reduction of the right hip because of incarcerated and rotated femoral head fragment (blue arrow) and presence of two small fragments one superior (red arrow) and the second inferior (green arrow), and small posterior wall fracture of left acetabulum (yellow star).

**Fig. 5:**
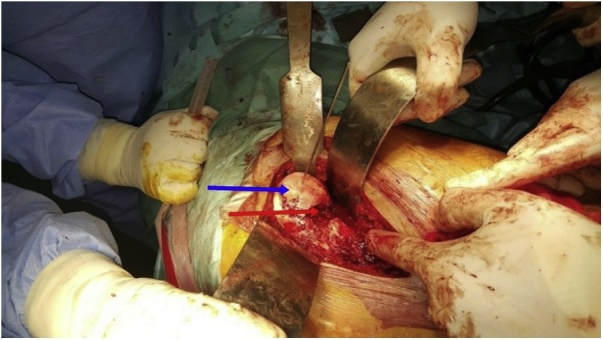
Hardinge approach to the right hip allowing osteosynthesis of Pipkin’s fracture with two Herbert screws.

**Fig. 6:**
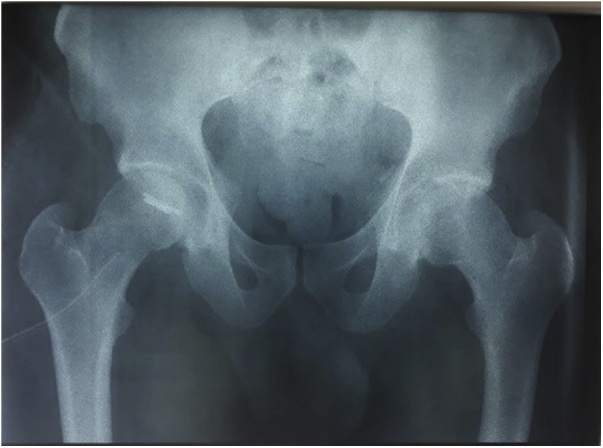
pelvic post-operative X-ray showing the Herbert screws fixation and good anatomic hip reduction.

**Fig. 7:**
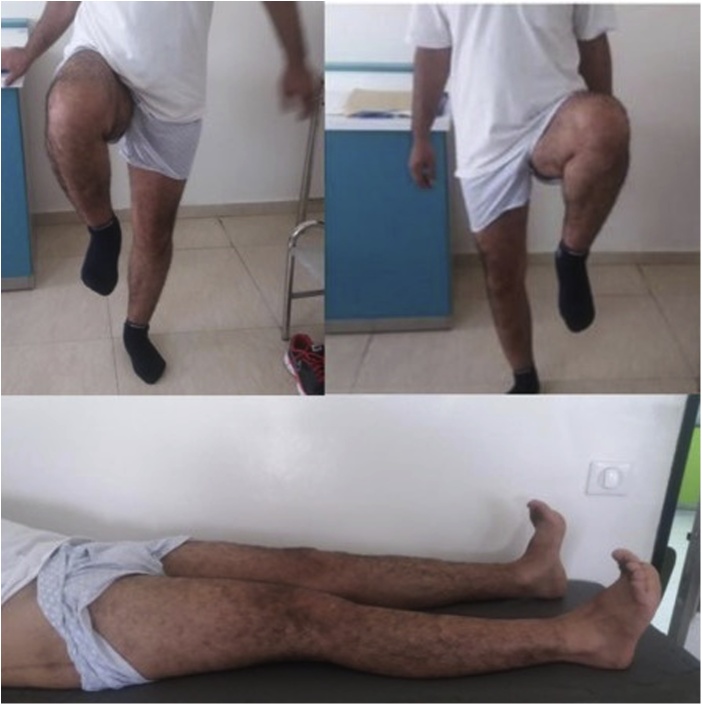
clinical examination of lower limbs and the skin incision of modified Hardinge approach.

**Fig. 8:**
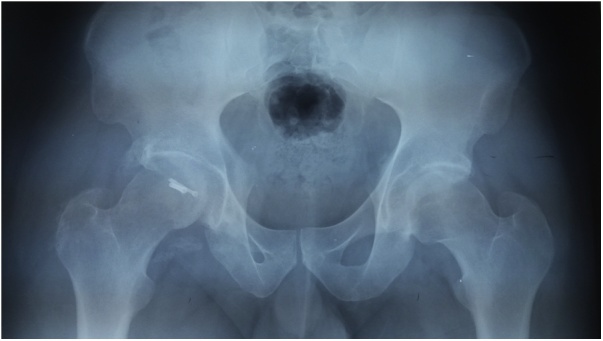
two years pelvic post-operative X-ray showing absence of osteoarthritis or avascular necrosis signs, but a right hip non-bridging Brooker type I heterotopic ossification was noticed.

A 40 years old man, was injured in a car accident as a front passenger, occurred during a frontal collision with another vehicle passing illegally in continuous white line down the center of a national road, the impact speed was 100 km per hour. Unfortunately, the patient was not restrained by seatbelt and sustained lower extremity and head impacts without loss of consciousness, he experienced a severe pain in his hips. On presentation to the emergency department, patient was hemodynamically stable and conscious, examination of the chest and abdomen was normal. However, both hips were deformed in flexion, internal rotation and adduction (Fig. 1), without neurovascular deficit or skin lesions of the lower limbs. Radiographs displayed bilateral posterior hip dislocation combined with right femoral head fracture, and small posterior wall fracture of the left acetabulum. (Fig. 2) Prompt hips reduction was performed within one hour of presentation through closed manipulation below general anesthesia, curarisation and controlled by fluoroscopy, patient was maintained in supine position on the ground and the aide applied a hand pression on the iliac wings of the pelvis, while the operator exerted a traction on the leg then flexed the hip with adduction and external rotation, the same technique was applied successfully to the second hip. The Left hip was stable up to 100° flexion and 45° internal and external rotation after reduction. However, the right hip was unstable, for this reason, we have positioned a posterior knee splint for temporary stabilization. Reduction of each hip was checked by antero-posterior pelvic radiograph (Fig. 3) and CT-scan (Fig. 4). X-ray showed an irreducible right femoral head fracture, but in the other side it showed a concentric reduction of the left hip joint. CT scan showed in the right side a one-third of femoral head suprafoveal fracture Pipkin type II that was anterior, rotated and incarcerated associated with two small fragments; one was superior and the second inferior. In the left side we have detected a minor posterior wall fracture of the left acetabulum without any intra-articular fragments. Surgical treatment was planned next day to accomplish anatomic open reduction of the right femoral head fracture and its internal fixation (ORIF) using a modified Hardinge approach that was chosen because the Pipkin II fragment was switched, therefore its reduction need surgical dislocation of the hip and a wide access to the femoral head. Moreover, this approach is known to have less risk of sciatic nerve injury, preserve the pelvitrochanteric muscles and have less risk of limping in comparison with the conventional Hardinge approach, because we spread the fibers of the gluteus medius just at its anterior middle one-third, preserving a great portion of this muscle insertions and function [1]. In the operational room, under general anesthesia the patient was placed in the full lateral position with pubic and sacral support, left leg was maintained in extension to stabilize ipsilateral hip, then a modified external approach of Hardinge was performed exposing the fascia lata that was incised in line with skin incision and retracted anteriorly while the gluteus maximus was retracted posteriorly to expose the common tendon of vastus lateralis and the gluteus medius, it was split longitudinally at the anterior third and sharply separated from the greater trochanter. Without extension more than 3 cm proximally to the insertion of the great trochanter to avoid inferior branch of the superior gluteal nerve injury. An anterior flap was formed by the anterior portion of the gluteus medius, the underlying gluteus minimus, and the anterior portion of the vastus lateralis. A T-shaped capsulotomy was performed to release the anterior capsule. The hip joint was then dislocated, and the femoral head fracture was exposed, we have explored a rotated pipkin II fragment and extracted two small parts that had footprints in the not weight bearing area in the distal part of the femoral head (Fig. 5). After cleaning and washing hip joint, we have reduced the Pipkin fragment and fixed them by two small pins that was over-drilled with a cannulated drill bit, followed by osteosynthesis with two Herbert screws, then hip was reduced and the flaps was repaired by transosseuses sutures to the great trochanter layer by layer using a vicryl number 2. Postoperative examination revealed no neurological deficit and the postoperative imaging of the pelvis showed an anatomic reduction of the femoral head fracture and a good position of the two Herbert screws (Fig. 6). The patient was kept on bed rest for three weeks, followed by a further six weeks of right non-weight bearing. At 6 months there was a good fracture healing, the patient had no limited hip motion, without limping and normal return to his daily activity (Fig. 7). Then at one and two years follow up there was no signs of osteoarthritis or avascular necrosis detected on the pelvic X-rays. However, a right hip non-bridging Brooker type I heterotopic ossification was noticed (Fig. 8).

## 3 Discussion

Bilateral posterior hip dislocation whether it's isolated or associated to the femoral head fracture is not common requiring a hight force to occur, the mechanism is a dashboard injury through adduction, internal rotation of lower limbs and flexed hips [2,3]. Therefore, we must inspect for other combined injuries, such as thoracic, abdominal or traumatic brain injury, which may lead to life-threatening conditions.

X-rays and CT scan evaluation were necessary to define the exact femoral head fracture dislocation pattern and to choose an ideal surgical approach.

Thompson classified posterior hip dislocation into 5 types depending on the associated fracture, type I is associated with or without a small fracture, type II is associated with a large single fracture of the posterior acetabular rim, type III with comminution of the acetabular ring, type IV with a fracture of the acetabular floor, the fifth type is associated with femoral head fracture, this last entity was later subdivided by Pipkin into 4 types: type I, fracture inferior to fovea; type II, fracture superior to fovea; type III, fracture of both femoral head and neck; type IV, fracture of femoral head and acetabulum, usually the posterior wall [4,5].

Other classification systems were described to illustrate the femoral head fractures such as the Brumback classification, the OTA (Orthopaedic Trauma Association) and the Chiron classification, but the Pipkin classification is probably the best known and the most used in scientific papers [[5], [6], [7]]. In our case it was an irreducible Pipkin II femoral head fracture treated by open reduction and internal fixation.

It is an orthopaedic emergency, closed reduction must be carried out within 6 h postinjury. Delay in the reduction of a dislocated hip joint increases the risk of sciatic nerve palsy and the incidence of osteonecrosis, which develops in 9% of hip fracture dislocations [7].

There is a lot of controversy concerning the surgical management of Pipkin’s fractures, there is increased risk of coxarthrosis after conservative treatment versus operative one, however there is no difference in AVN risk. Many authors have recommended a surgical treatment for pipkin II and Pipkin I fracture if the fragment is large, even though it is located under the fovea, the fragment should be fixed, because it is intraarticular to avoid coxarthrosis. Some other ones recommend orthopedic treatment if the fracture fragment is reduced by closed means and if the hip joint is congruous and stable, Concerning the Pipkin III and IV fractures, they are treated conservatively through internal osteosynthesis or first intention hip arthroplasty [8,9].

We believe that if there are no vital contraindications, all Pipkin fractures should be operated by reducing bigger fragments and extracting smaller ones in Pipkin I and II fractures, try to preserve the femoral heads in young patients with Pipkin III fractures by osteosynthesis and for elderly patients with Pipkin III or IV fractures, primary arthroplasty is the best choice.

Various operative approaches have been used for open reduction and internal fixation of Pipkin fractures, it depends on direction of the dislocation, associated acetabular fractures and the position of the femoral head fragment that can be anterior, posterior or medial. Through literature publications many approaches were used essentially posterolateral (Moor), anterolateral (Watson-Jones), iliofemoral (smith-Petersen), and more recently lateral (Hardinge) or anterior approaches (Heuter). While the Kocher Langenbeck, and the posterior approaches with trochanteric flip osteotomy were recommended by the earliest authors, their main arguments was the notion of traumatic posterior capsular lesion, and the presence of associated fracture of the posterior wall, posterior or transverse column of the acetabulum [2,7].

Our preference for the modified direct lateral approach to treat Pipkin II fracture was justified by the wide access to the femoral head fracture through this approach and the preservation of the pelvitrochanteric muscles and sciatic nerve. However, precautions should be taken in the lateral position to avoid contralateral hip re-dislocation by maintaining the contralateral leg in extension.

Arthroscopy is an alternative and efficient emerging technique for extracting small intra-articular fragments [10].

Herbert or cancellous screws are most used, recently we found many other materials such as bioabsorbable screws, biodegradable polylactide pins, and osteochondral autografts for the treatment of femoral head defects [11].

Whatever the surgical technique or the approach used, complications are not inevitable and must be searched periodically, the main complications are avascular necrosis of the femoral head, heterotopic ossification [12], and posttraumatic osteoarthritis.

## 4 Conclusion

Posterior dislocated hip is a rare injury, it should have a closed reduction within 6 h, aiming at anatomic restoration of joint congruity. CT-scan should be systematic to determine femoral head fractures pattern and surgical approach.

A good final outcome does not necessarily follow a specific approach, but depends on the initial injury and subsequent treatments.

## Sources of funding

All authors disclose that this manuscript didn’t received no specific grant from any funding agency.

## Ethical approval

The study is exempt from ethnical approval in our institution. This is a case report and the patient give us informed consent for publication.

## Consent

Patient gives informed consent for publication.

## Author contribution

Abderrahim Zaizi, Hicham Ahmed Benomar and Talal Grimi make substantial contributions to acquisition of data, conception and design, and analysis and interpretation of data.

Monsef Boufettal and Mohamed S. Berrada participate in revising it critically for important intellectual content and give final approval of the version to be submitted.

## Registration of research studies

This case report don’t need to be registered because is not first-in-man.

## Guarantor

Abderrahim Zaizi and Hicham A. Benomar are the guarantor of this publication.

## Provenance and peer review

Not commissioned, externally peer-reviewed.

## Declaration of Competing Interest

The authors report no conflicts of interest. The authors alone are responsible for the content and writing of the paper.
